# The clinical influence of nasal surgery on PAP compliance and optimal application among OSA subjects uncomfortable with PAP device wear

**DOI:** 10.1038/s41598-023-31588-7

**Published:** 2023-03-16

**Authors:** Hyunkyung Cha, Heonjeong Oh, Sun A Han, Seo Young Kim, Jeong Kyou Kim, Hae Chan Park, Doo Hee Han, Dong-Young Kim, Hyun Jik Kim

**Affiliations:** 1 Department of Otorhinolaryngology, Seoul National University College of Medicine, 103 Daehak-ro, Jongno-gu, Seoul, 03080 Republic of Korea; 2 Department of Otorhinolaryngology-Head and Neck Surgery, Soonchunhyang University Cheonan Hospital, Soonchunhyang University College of Medicine, Cheonan, Republic of Korea; 3 Department of Otorhinolaryngology-Head and Neck Surgery, Dongtan Sacred Heart Hospital, Hallym University College of Medicine, Hwaseong, Republic of Korea; 4 Sensory Organ Research Institute, Seoul National University Medical Research Center, Seoul, Republic of Korea

**Keywords:** Outcomes research, Translational research

## Abstract

This study aimed to evaluate the alteration of PAP compliance after nasal surgery and to determine the optimal indications of nasal surgery in obstructive sleep apnea (OSA) subjects. Among OSA subjects using PAP devices, 29 subjects who underwent septoturbinoplasty due to nasal obstruction were included and their pre- and postoperative medical and PAP records were reviewed retrospectively. Postoperative autoPAP usage data was further assessed by grouping the compliance (the percentage of days with usage ≥ 4 h) data (group 1: the good compliance group; group 2: the poor compliance group). The data showed that 56% of subjects in group 1 complained of nasal obstruction as the only barrier to using a PAP device and about 89% reported experiencing the efficacy of PAP usage. Both the mean and peak average PAP pressures were significantly reduced in group 1 following nasal surgery. Group 2 had multiple subjective problems that interfered with wearing a PAP device and reported a lack of experiencing the efficacy of PAP usage. Preoperative nasal cavity volume values were smaller and absolute blood eosinophil counts were significantly lower in group 1. The current data demonstrate that nasal surgery might increase the compliance of PAP device wear in OSA subjects who complained of only nasal obstruction as a barrier to wearing PAP and who had small nasal cavity volumes combined with allergic inflammation.

## Introduction

Obstructive sleep apnea (OSA) is a breathing disorder caused by the narrowing of the multi-level upper airway that interrupts normal ventilation during sleep^1–4^. The nose is the starting point of the airway system, and about 50–70% of all airway resistance is devoted to the nasal cavity^2^. If there is no pathological problem, people typically breathe through their nose while sleeping^3^. It is well known that nasal obstruction worsens the OSA, and its removal reduces OSA severity^3,4^.

Positive airway pressure (PAP) is the treatment of choice for moderate-to-severe OSA patients^5^. PAP therapy can relieve subjective symptoms and prevent life-threatening conditions^5–10^. However, compliance rate with PAP therapy can be low, with reported rates of 54–75%^11^. The American Academy of Sleep Medicine has recommended that PAP therapy for adult OSA patients be initiated using either by autoPAP therapy or in-laboratory titration PAP therapy, based on the patient circumstances^12^. Its guidelines also emphasize the need to identify clinical factors that can reduce compliance rates and correct them to ensure adequate treatment and compliance with PAP therapy^12,13^.

The most frequent complaints of PAP non-compliance patients include an inconvenience associated wearing, chest discomfort, allergic reaction, mouth dryness, and difficulty in breathing during sleep^14^. Although automatic PAP (autoPAP) adjusts the pressure based on inhalation and works in a specific pressure range compared to continuous PAP, our previous data showed that about 9% of OSA subjects with poor compliance complained of nasal obstruction as a main problem interfering with wearing the PAP device^14,15^. Another study also reported that autoPAP therapy intolerance according to nasal problems seems to account for approximately 12% of cases^16^. The nasal obstruction seems to have a decisive effect on the reduction of the compliance rate if this problem is not overcome in the early stages of using a PAP^17^.

The presence of a narrow nasal cavity is believed to contribute to an increase in therapeutic PAP and subjective discomfort, ultimately leading to poor PAP compliance^14,18^. Furthermore, some reports state that sleep apnea surgery for PAP intolerant patients can make PAP a therapeutic option, and, in particular, PAP compliance may improve following nasal and upper airway surgery^19–21^. One study suggested that subjective nocturnal nasal obstruction is not a predictor of becoming a non-user of PAP within the first 2 years^22^. However, this study has also found that a small nasal cavity volume at baseline may be a determinant of non-compliance^22^. According to the Poiseuille’s law, airway resistance is critically determined to the fourth power of airway radius^23^. More, the Bernoulli principle implies that the increased airflow velocity by the nasal blockage decreases the static pressure in the pharyngeal airway and makes the pharyngeal wall to collapse as a downstream segment^23,24^. Altogether, the treatment for nasal pathology may be a crucial treatment strategy for patients with OSA. But still, the therapeutic effect of correction for nasal obstruction on the compliance of PAP remains controversial or rather confusing due to a lack of adequate objective outcome measurements^11,15,25–27^.

In the present study, we aimed to examine factors affecting compliance to autoPAP after surgical correction of nasal obstruction in OSA subjects and also sought to determine whether surgical correction of nasal obstruction would improve PAP compliance in OSA subjects with nasal pathologies based on PAP parameters.

## Methods

### Ethical approval

The institutional review board (IRB) of Seoul National University Hospital approved this study (IRB No. 2206–085-1332). All methods were performed in accordance with the approved guidelines and the Declaration of Helsinki. All personal information was kept confidential as required. Informed consent was waived because of the retrospective nature of the study.

### Study design and subjects

A retrospective medical review of patients who were diagnosed with OSA and prescribed autoPAP between January 2017 and July 2021 at the Seoul National University Hospital was performed. Subjects complaining of snoring and apnea underwent endoscopic examination, polysomnography, and drug-induced sleep endoscopy (DISE). OSA was diagnosed if the apnea–hypopnea index (AHI) value was > 15 or > 5 with related symptoms or problems, including daytime sleepiness, declined cognitive function, mood disorder, hypertension, a history of infarction, and decreased O_2_ saturation of < 85%. Severity was classified according to the AHI; mild OSA was defined as an AHI value of 5–14, moderate OSA was defined as an AHI value of 15–30, and severe OSA was defined as an AHI value of > 30. AutoPAP therapy was prescribed for patients with OSA. The autoPAP usage records were reviewed once every 3 months. The prescribed autoPAP device was initially set in the range of 5–12 mmHg and adjusted according to the patient’s follow-up autoPAP usage reports. The patients who complained of nasal obstruction as a barrier to using a PAP device with a deviated nasal septum who underwent nasal surgery were included in this study. Patients with severe comorbid diseases (such as cancer), diseases that decrease O_2_ saturation (such as congestive heart failure and chronic obstructive pulmonary disease), and central sleep apnea who were prescribed alternative treatment to CPAP were excluded. Additionally, patients who underwent oropharynx sleep surgery and nasal surgery at the same time or had loss of follow-up were also excluded. The flowchart of study design is indicated in Supplementary Fig. 1.

### Surgical procedure

The indication for septoturbinoplasty was determined to be a grade 2 or higher deviated nasal septum with the complaint of nasal obstruction^28^. The volume reduction for both sides of the inferior turbinate were performed with a COBLATOR™ II surgery system (Smith & Nephew, London, UK). The autoPAP was worn again 1 week after the nasal surgery.

### Outcome assessment

Patients visited outpatient clinic once every 3 months to review the usage records of autoPAP. In order to check psychosocial barriers related to PAP usage, we asked two questions of all patients: (1) Have you experienced any efficacy while using PAP? (2) Have you encountered any difficulties or complications while using the PAP device? If any difficulties were reported, we also asked for the reasons.

AutoPAP usage data before and after the surgery were assessed. The pairwise comparison of the percentage of days with device usage, the average use time, the percentage of days with usage ≥ 4 h, the mean pressure, the peak average pressure, the average device pressure ≤ 90% of the time, and AHI values before and after the surgery was performed.

**Figure 1:**
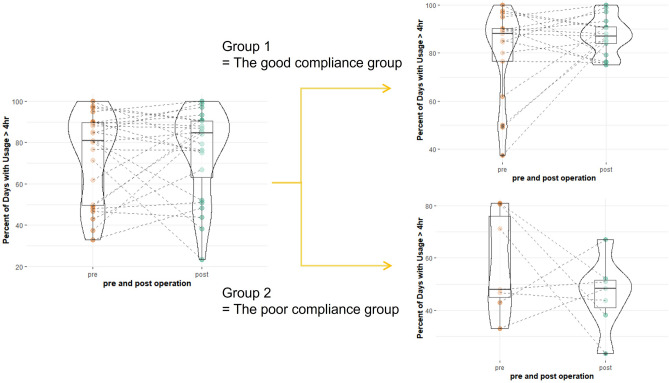
The autoPAP compliance groups were classified according to the percentage of days spent using PAP for > 4 h/day after surgery. The cutoff value was 70%.

Adequate PAP device compliance was defined as ≥ 4 h of administration for ≥ 70% of the nights (according to the U.S. Center for Medicare and Medicaid Services criteria)^29,30^. Postoperative autoPAP usage data was further assessed by grouping the compliance data. Group 1 was defined as the good compliance group, in which patients used PAP for at least 4 h per day, on 70% or more days, after surgery. Group 2 was defined as the poor compliance group, in which patients used PAP for at least 4 h per day, on fewer than 70% of days, after surgery. (Fig. 1).

The values of minimal cross-sectional area (MCA) and nasal cavity volume (NCV) from acoustic rhinometry are indicated as smaller values among the measures from both nasal cavities. The diagnosis of allergic rhinitis (AR) was made based on the patient history and laboratory data, including skin prick test, and serum-specific immunoglobulin (Ig) E levels. Perennial Allergic Rhinitis (AR) refers to cases of rhinitis caused by allergies to perennial allergens, such as dust mites, mold, pet dander, and cockroaches. Seasonal AR, on the other hand, refers to cases of rhinitis caused by seasonal allergens like tree, grass, and weed pollen. The blood eosinophil count was analyzed based on the highest value measured.

### Statistical analysis

Continuous variables are presented as mean ± standard deviation values. Continuous variables were analyzed by the *t* test or Mann–Whitney *U* test (two-tailed) depending on normality. Categorical variables were assessed by a chi-squared test. Paired samples were assessed by the Wilcoxon signed-rank test (two-tailed). Multivariate logistic regression analysis was performed with stepwise selection method. Log transformation was used for no normality assumption independent variables in logistic regression. *P* < 0.05 was considered to be statistically significant. Missing values were pairwise deleted. All statistical analysis was performed using R (version 4.1.2; R Foundation for Statistical Computing, Vienna, Austria) and GraphPad Prism (version 9.0; GraphPad Software Inc., La Jolla, CA, USA).

## Results

### Clinical characteristics of patients

Patient’s demographic characteristics are presented in Table 1. Twenty-nine OSA patients (All male; mean age 52.45 years) were included. Their Tonsil grade 0, I, II, III, or IV was observed in 7%, 62%, 28%, 3%, and 0% of patients, respectively. Palatal grade I, II, III, or IV was found in 0%, 3%, 48%, and 48% of patients, respectively. The mean preoperative AHI value was 50.19 ± 27.17/h, and the mean minimal SpO2 was 75 ± 11%. Nasal surgeries were all successful. All patients showed a significant improvement of nasal obstruction after septoturbinoplasty and patent nasal airway by intranasal endoscopic examination.

**Table 1:** Demographic data of the recruited OSA subjects.

Parameter	Values
Age (years)	52.45 ± 10.57
Sex	Male, 29 (100%); female, 0 (0%)
BMI (kg/m^2^)	28.57 ± 3.79
Preoperative PSG	
AHI (/h)	50.19 ± 27.17
Supine AHI (/h)	67.51 ± 32.26
Non-supine AHI (/h)	25.26 ± 27.61
RDI (/h)	51.05 ± 26.95
Minimum SpO_2_ (%)	75 ± 11
Severity (mild/moderate/severe)	3/6/20
Physical examination	
Tonsil grade (0/I/II/III/IV)	2/18/8/1/0
Palatal grade (I/II/III/IV)	0/1/14/14
Comorbidity	
HTN	20/29 (69%)
DM	6/29 (21%)
AR	14/28 (50%)
Lab	
Absolute eosinophil count (/µL)	258.42 ± 246.56
MCA (cm^2^)	0.49 ± 0.08
NCV (cm^3^)	6.07 ± 1.06

BMI, body mass index; PSG, polysomnography; AHI, Apnea–Hypopnea Index; RDI, respiratory disturbance index; SpO_2,_ pulse oximeter oxygen saturation; HTN, hypertension; DM, diabetes mellitus; AR, allergic rhinitis; MCA, minimal cross-sectional area; NCV: nasal cavity volume.

Variables were stated as numbers (%) or mean ± standard deviation values.

### AutoPAP use prior to and following nasal surgery

The changes in PAP parameters after nasal surgery are indicated in Table 2 and we did not find any significant change in all PAP parameters. The percentage of days with device usage pre-operation was 89 ± 13% and that post-operation was 85 ± 20% (*P* = 0.356). The average usage time of PAP was 329.24 ± 83.38 min prior to nasal surgery and 338.49 ± 66.09 min following surgery (*P* = 0.350). The percentages of days with usage for ≥ 4 h pre-operation and post-operation, respectively, were 74 ± 21% and 73 ± 24% (*P* = 0.979). The mean device pressure pre-operation was 801.18 ± 213.78 Pascal (Pa) in OSA subjects and 780.59 ± 179.46 Pa post-operation (*P* = 0.115). The peak average pressure pre-operation was 959.06 ± 202.01 Pa and post-operation was 937.49 ± 183.38 Pa (*P* = 0.137). The average device pressure ≤ 90% of the time was 948.28 ± 191.22 pre-operation and 928.66 ± 176.51 post-operation (*P* = 0.120). The average AHI values pre-operation and post-operation were 4.48 ± 4.32 /h and 4.72 ± 3.50/h (*P* = 0.560). These results suggest that nasal surgery does not induce changes in objective PAP use time or PAP pressure in a simple comparison when OSA subjects using autoPAP devices complain of nasal obstruction.

**Table 2:** AutoPAP usage data in OSA subjects.

Parameter	Pre-operation	Post-operation	*P*
Percentage of days with device usage (%)	89 ± 13	85 ± 20	0.356
Average usage (min)	329.24 ± 83.38	338.49 ± 66.09	0.350
Percentage of days with usage ≥ 4 h (%)	74 ± 21	73 ± 24	0.979
Mean pressure (Pa)	801.18 ± 213.78	780.59 ± 179.46	0.115
Peak average pressure (Pa)	959.06 ± 202.01	937.49 ± 183.38	0.137
Average device pressure ≤ 90% of the time (Pa)	948.28 ± 191.22	928.66 ± 176.51	0.120
Average AHI (/h)	4.48 ± 4.32	4.72 ± 3.50	0.560

AHI, Apnea–Hypopnea Index.

### Compliance with autoPAP therapy in OSA subjects with nasal surgery according to psychosocial barriers

Eighteen subjects were included into group 1. Of these, 56% complained of nasal obstruction as their only barrier to using a PAP device (Table 3). In contrast, 44% had multiple problems (including frequent business trips, difficulty lying on one’s side, oropharynx air leakage, discomfort with wearing a mask, and dry nose) that interfered with wearing a PAP device accompanied by nasal obstruction. About 89% were reported to be satisfied with the efficacy of PAP therapy due to the improvement of cardiac arrhythmia, voice change, dry mouth, sputum, excessive daytime sleepiness, headache, and/or fatigue. However, 11% in group 1 did not feel there were any meaningful changes in subjective symptoms when using their autoPAP device.

**Table 3:** Subjective factors related to interference with wearing a PAP device in OSA subjects.

PAP usage barriers	Patients		*P*
	**Group 1 (n = 18)**	**Group 2 (n = 11)**	
None other than nasal resistance	10 (56%)	0 (0%)	0.008*
Frequent business trips	1 (6%)	2 (18%)	0.649
Difficulty lying on one’s side	3 (17%)	1 (9%)	0.985
Oropharynx air leakage	4 (22%)	2 (18%)	1.000
Troublesome cleaning the device	0 (0%)	1 (9%)	0.800
Discomfort with wearing a mask	1 (6%)	6 (55%)	0.011*
Dry nose	1 (6%)	0 (0%)	1.000
Uncontrolled allergic rhinitis symptoms	0 (0%)	4 (36%)	0.028*
Rhinitis medicamentosa	0 (0%)	1 (9%)	0.800
A lack of experiencing efficacy	2 (11%)	8 (73%)	0.003*

PAP, positive airway pressure.

*Statistically significant (*P* < 0.05).

Eleven subjects who were classified into group 2, and they reported multiple problems (including frequent business trips, difficulty lying on one’s side, oropharynx air leakage, troublesome cleaning the device, discomfort with wearing a mask, uncontrolled allergic rhinitis symptoms, and rhinitis medica mentosa) that interfered with wearing the PAP device (Table 3). None of these subjects reported nasal obstruction as the only obstacle to PAP usage. About 27% were reported to be satisfied with the efficacy of PAP due to improvements in dry mouth, daytime sleepiness, and headache. Through comparative analysis between groups, we found that OSA subjects who reported only nasal obstruction as a barrier to PAP use were all group 1 patients. On the other hands, OSA patients in group 2 reported multiple complaints as barriers to PAP use (*P* = 0.008). The number of patients who experienced no usefulness of the PAP device was significantly higher in group 2 (*P* = 0.003). The numbers of patients feeling discomfort wearing a mask and uncontrolled AR symptoms were also significantly higher in group 2 (*P* = 0.011 and *P* = 0.028, respectively). Interestingly, the sleep quality was dramatically improved in 3 subjects who were classified into group 2. Among them, 2 subjects underwent postoperative PSG and 1 patient improved their AHI value from 70.6 to 25.0/h, while another improved their value from 43.3 to 16.7/h. Although we recommended these subjects should continue using PAP, despite nasal surgery being effective in improving their nasal pathologies, they were satisfied with the improvement in sleep quality and refused to use the PAP device anymore.

These results revealed that nasal surgery can be effective in OSA subjects who are uncomfortable wearing a PAP device due to only nasal obstruction, and the compliance rate after surgery was high. However, the nasal surgery did not have much of an effect in OSA subjects who complained of multiple problems.

### Comparison of PAP parameters between the PAP compliance groups

We evaluated the PAP parameters of OSA patients who had undergone nasal surgery, based on PAP compliance (Table 4). However, we excluded 2 patients who refused to wear the PAP device, as they had experienced marked improvement in their subjective symptoms and sleep parameters.

**Table 4:** AutoPAP usage data of the PAP compliance groups following nasal surgery.

Parameter	Group 1 (n = 18)			Group 2 (n = 9)		
	Pre-operation	Post-operation	*P*	Pre-operation	Post-operation	*P*
Percentage of days with device usage (%)	94 ± 7	94 ± 6	0.751	75 ± 16	70 ± 25	0.688
Average usage (min)	351.96 ± 72.51	369.76 ± 52.80	0.211	277.50 ± 91.59	288.31 ± 39.46	0.773
Percentage of days with usage ≥ 4 h (%)	80 ± 19	87 ± 8	0.353	58 ± 20	46 ± 13	0.375
Mean pressure (Pa)	780.22 ± 236.68	738.38 ± 183.79	0.013*	876.99 ± 145.77	907.82 ± 102.21	0.453
Peak average pressure (Pa)	938.45 ± 227.89	898.75 ± 195.43	0.049*	1048.09 ± 116.09	1062.80 ± 85.48	0.844
Average device pressure ≤ 90% of the time (Pa)	920.67 ± 212.88	891.83 ± 188.39	0.062˚	1038.10 ± 102.29	1043.71 ± 74.01	0.688
Average AHI (/h)	4.56 ± 5.17	4.12 ± 3.76	0.901	4.33 ± 2.35	5.73 ± 2.97	0.383

AHI, Apnea–Hypopnea Index.

*Statistically significant (*P* < 0.05).

**Figure 2:**
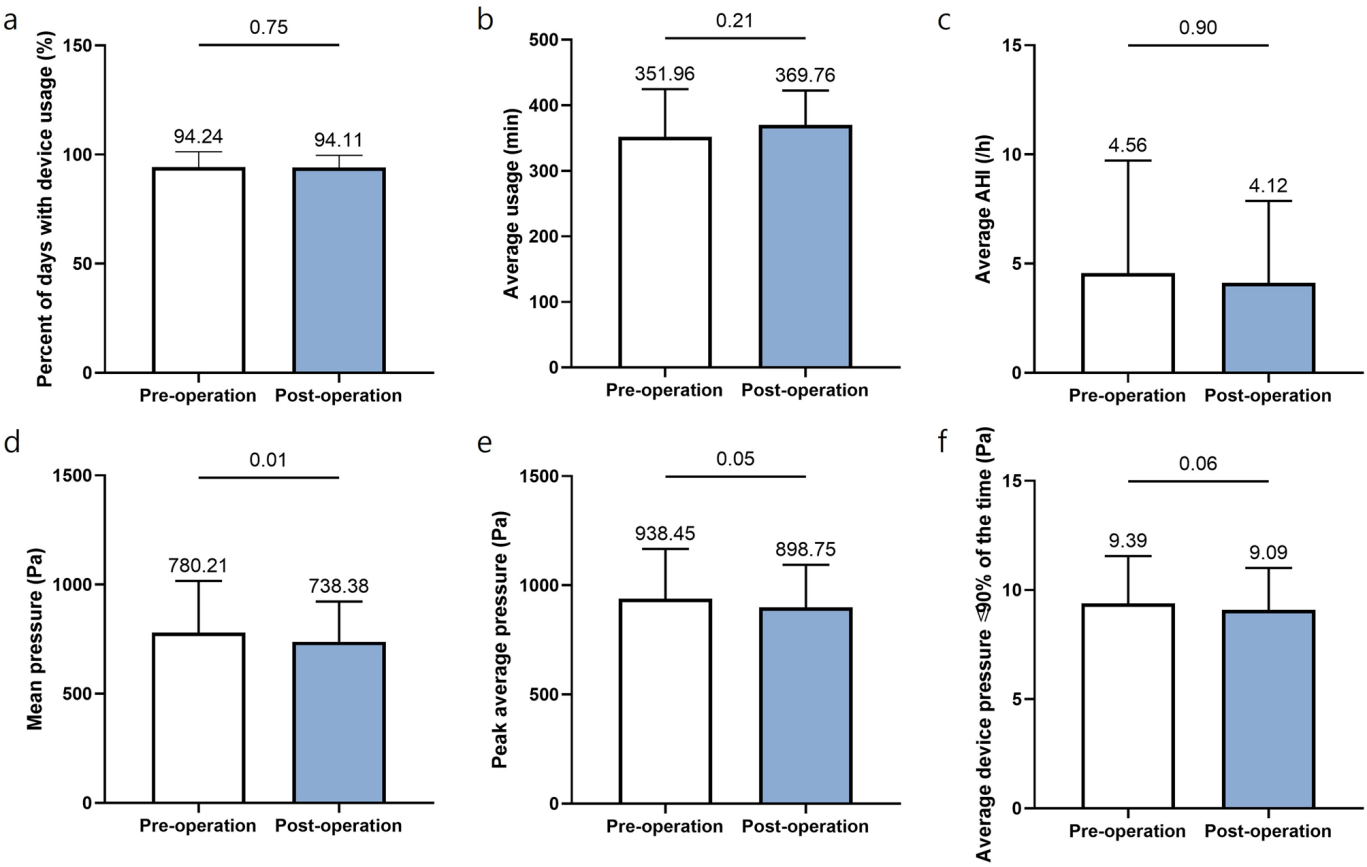
AutoPAP usage data in group 1. The average values of mean and peak average pressures (Pa) significantly changed after nasal surgery. (**a**) Percentage of days with device usage (%). (**b**) Average usage (min). (**c**) Average AHI (/h). (**d**) Mean pressure (Pa). (**e**) Peak average pressure (Pa). (**f**) Average device pressure ≤ 90% of the time (Pa).

**Figure 3:**
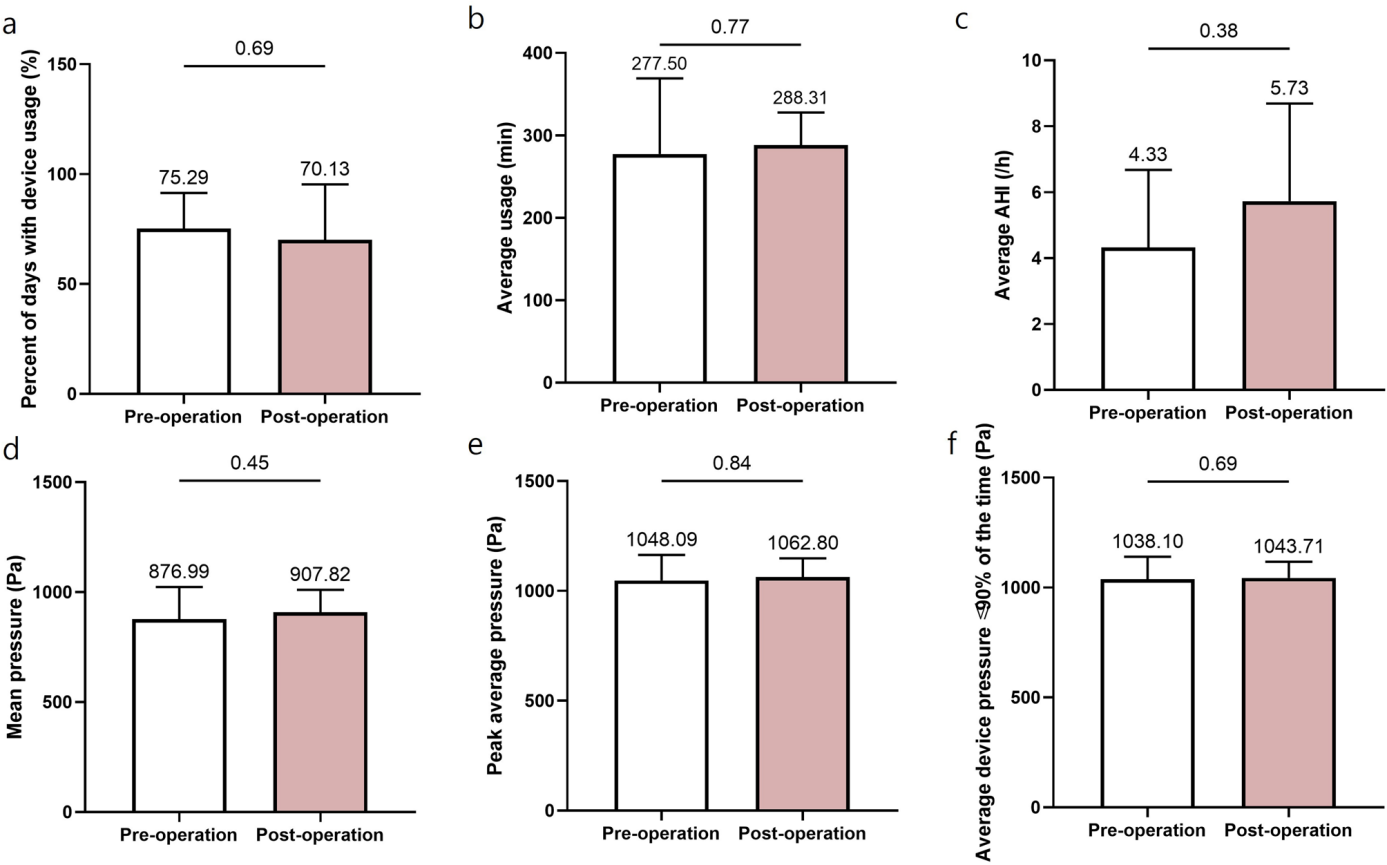
AutoPAP usage data in group 2. There were no significant changes in the AutoPAP values postoperatively. (**a**) Percentage of days with device usage (%). (**b**) Average usage (min). (**c**) Average AHI (/h). (**d**) Mean pressure (Pa). (**e**) Peak average pressure (Pa). (**f**) Average device pressure ≤ 90% of the time (Pa).

Figure 2 indicates group 1 PAP parameters. The PAP records showed that the percent of days with PAP use before nasal surgery was 94 ± 7% and that after surgery was 94 ± 6%, while the average time of PAP usage before surgery was 351.96 ± 72.51 min and that after the surgery was 369.76 ± 52.80 min. Interestingly, the mean PAP pressure value was 780.22 ± 236.68 Pa but decreased significantly to 738.38 ± 183.79 Pa after surgery (*P* = 0.013). The peak average pressure value was also significantly reduced from 938.45 ± 227.89 Pa to 898.75 ± 195.43 Pa after surgery (*P* = 0.049). In addition, the average device pressure ≤ 90% of the time, which was 920.67 ± 212.88 Pa, changed to 891.83 ± 188.39 Pa after nasal surgery in group 1 (*P* = 0.062). We did not observe any significant alterations in PAP parameters, including the time of PAP use and PAP pressure, in group 2 depending on nasal surgery (Fig. 3 and Table 4). Based on these findings, we estimated that PAP device wear after nasal surgery had an effect on the surgical result in OSA subjects, and PAP pressure was actually less in these OSA subjects.

### Differences in demographic factors among the PAP compliance groups

**Figure 4:**
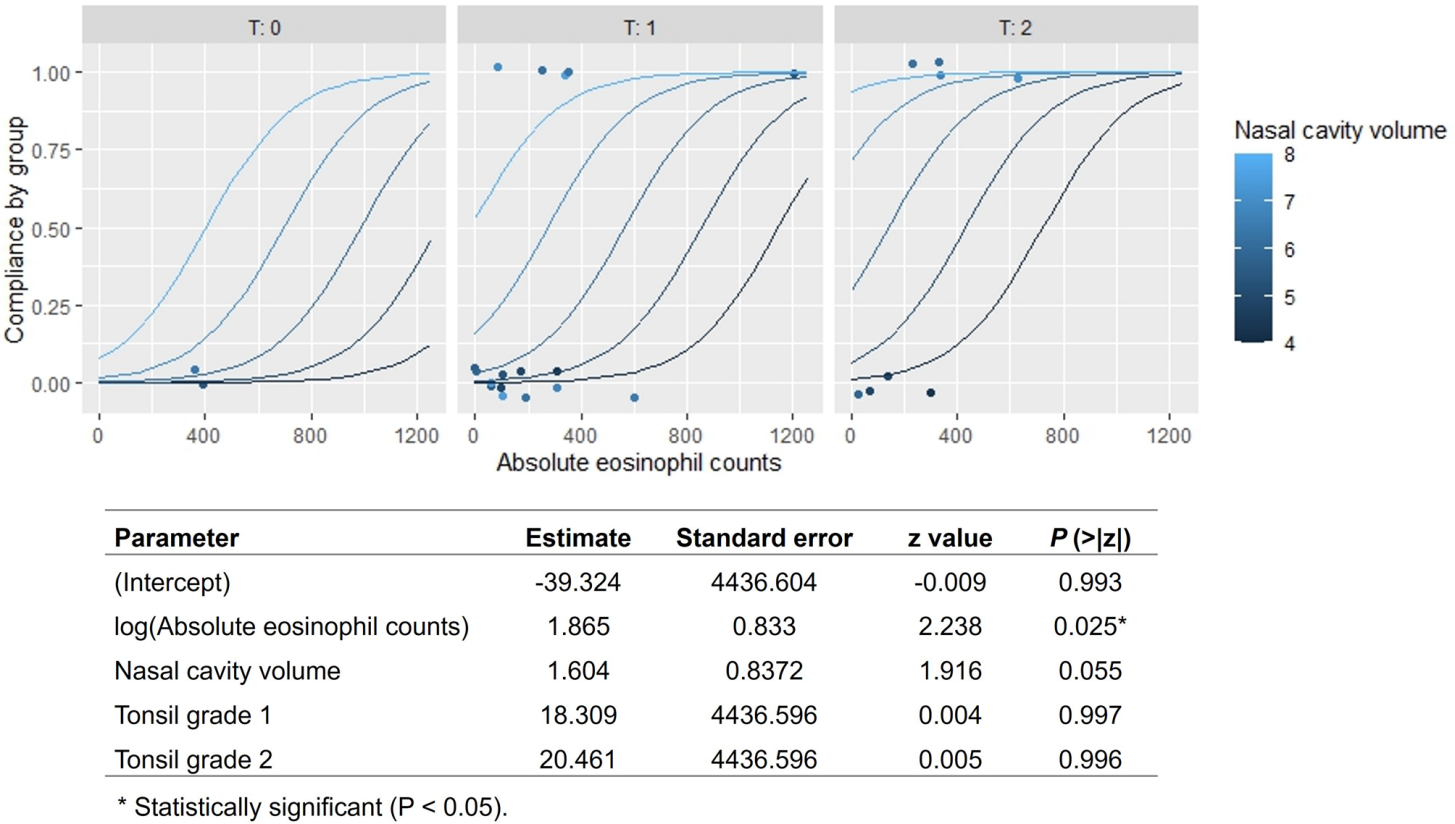
Prediction of the compliance group by the multiple logistic regression model. The y-axis with a value of 0 refers group 1 and a value of 1 refers to group 2. T denotes Tonsil grade.

In order to identify potential factors affecting PAP compliance in conjunction with nasal obstruction, we compared demographic data, such as age, sex, BMI, preoperative PSG, physical examination findings, comorbidities, acoustic rhinometry, and blood eosinophil count between the PAP compliance groups (Table 5). We did not find any significant differences in age, BMI, PSG parameters, grade of oropharyngeal structures, or comorbid diseases (including AR) between groups. However, the mean preoperative nasal cavity volume of group 1 (5.74 ± 0.96 cm^3^) was significantly less than that of group 2 (6.53 ± 0.69 cm^3^), and absolute blood eosinophil count was also significantly lower in group 1 (182.06 ± 163.00/μL) than group 2 (427.40 ± 349.12/μL) (*P* = 0.045 and *P* = 0.030, respectively). The preoperative MCA of group 1 was relatively smaller (0.48 ± 0.09 cm^2^) than that of group 2 (0.52 ± 0.04 cm^2^) (*P* = 0.113). The multiple logistic regression model was applied using independent variables from Table 5. Results indicate that blood eosinophil count (*P* = 0.025) and nasal cavity volume (P = 0.055) are potential factors that may contribute to an increased compliance rate following nasal surgery. These findings are supported by the data presented in Fig. 4.

**Table 5:** Demographic differences between the PAP compliance groups.

Parameter	Group 1 (n = 18)	Group 2 (n = 9)	*P*
Age (years)	54.33 ± 10.56	48.33 ± 11.20	0.189
BMI (kg/m^2^)	27.58 ± 3.53	30.01 ± 4.19	0.131
Preoperative PSG			
AHI (/h)	52.20 ± 28.16	44.71 ± 28.25	0.631
Supine AHI (/h)	67.45 ± 30.84	67.83 ± 38.63	0.668
Non-supine AHI (/h)	20.24 ± 20.23	34.89 ± 39.12	0.657
Severity (mild/moderate/severe)	1/5/12	2/1/6	0.325
RDI (/h)	51.72 ± 24.42	51.17 ± 33.06	0.980
Minimum SpO_2_ (%)	74 ± 12	74 ± 10	0.733
Average SpO_2_ (%)	91 ± 7	90 ± 4	0.107
Physical examination			
Tonsil grade (0/I/II/III/IV)	2/12/4/0/0	0/5/4/0/0	0.347
Palatal grade (I/II/III/IV)	0/0/9/9	0/1/4/4	0.354
Comorbidity			
HTN	12 (67%)	7 (78%)	0.551
DM	4 (22%)	2 (22%)	1.000
AR	7 (41%)	5 (56%)	0.484
Single/multiple Sensitizations	4/3	1/4	0.198
Perennial/seasonal AR	4/3	0/5	0.147
Lab			
Absolute eosinophil count (/µL)	182.06 ± 163.00	427.40 ± 349.12	0.030*
MCA (cm^2^)	0.48 ± 0.09	0.52 ± 0.04	0.113
NCV (cm^3^)	5.74 ± 0.96	6.53 ± 0.69	0.045*

BMI, body mass index; PSG, polysomnography; AHI, Apnea–Hypopnea Index; RDI, respiratory disturbance index; SpO_2_, pulse oximeter oxygen saturation; HTN, hypertension; DM, diabetes mellitus; AR, allergic rhinitis; MCA, minimal cross-sectional area; NCV: nasal cavity volume.

Variables are stated as numbers (%) or mean ± standard deviation values.

*Statistically significant (*P* < 0.05).

## Discussion

The narrow airway anatomy and the complaint of difficulty exhaling against high pressure are important factors affecting compliance with CPAP^15,31,32^. Our previous study also showed that correction of nasal pathologies and relief of nasal obstruction can improve sleep parameters in OSA subjects, leading to better sleep quality^3^. As a result, clinicians often consider method to improve nasal breathing in OSA patients who complain of nasal obstruction, including medication and nasal surgery, when PAP therapy is necessary to control their OSA-related symptoms^33,34^. Technological development in PAP devices are also aimed at reducing the mask pressure during expiration^27^. However, in our data, we did not observe a significant change in objective PAP use time or PAP pressure for OSA subjects using autoPAP devices after nasal surgery, which is consistent with previous studies using CPAP with a reduction of expiratory pressure^27,35^. Despite decades of research, it is noteworthy that there is no single factor that reliably predicts PAP compliance due to complicated barrier dynamics^36^. A combination of biomedical and psychological predictors has been found to have the best predictive power for explaining PAP compliance^36^.

Our data showed that surgical correction of nasal pathologies improved the PAP pressure and increased the rate of compliance with autoPAP therapy in OSA subjects who complained of nasal obstruction as the main barrier to PAP device use. Värendh et al.^22^ previously reported that subjective nasal obstruction is not a predictor of poor continuous PAP compliance within the first two years. However, they did not confirm the presence of any co-subjective barriers^22^. Poirier et al.^34^ reported increased PAP compliance after nasal surgery among PAP users with nasal obstruction as the sole stated non-compliance factor. However, OSA subjects with other concomitant barriers to PAP device use may not respond to nasal surgery and their compliance did not improve even after their nasal obstruction was completely resolved. Furthermore, if an OSA subject experienced high efficacy PAP use, the subject seemed to be more likely to show a good compliance after nasal surgery. Our data revealed that OSA subjects who complained of discomfort wearing a mask and a lack of PAP efficacy did not respond to nasal surgery and were included into group 2, even though their nasal obstruction had improved. For example, two patients had multiple barriers to using PAP (both complained of discomfort while wearing a mask) in group 2 following nasal surgery. Specifically, these two OSA subjects underwent nasal surgery, and their nasal breathing improved too much, accompanied by a markedly improved OSA severity and quality of sleep. As a result, they did not try to wear a PAP device after nasal surgery and were included into group 2. These cases show that, even if nasal surgery is successful enough to resolve the nasal obstruction and OSA subjects do not want to wear the PAP device anymore, it is important for clinicians to conduct close communication with OSA subjects to identify OSA-related problems and to provide medical advice about the necessity and efficacy of PAP therapy.

Considering the differences in demographic parameters between the PAP compliance groups, the significant reduction in PAP pressure exhibited in group 1 and the presence of smaller nasal cavity volumes and lower absolute blood eosinophil counts showed better compliance with PAP device use after nasal surgery. A previous study also showed a small nasal cavity volume as a determinant of becoming a non-user of PAP after 2 years^22^. Based on these findings, we believe that it is important to evaluate the patient's discomfort accurately and measure their nasal volume and blood eosinophil count before wearing the PAP device in order for nasal surgery to improve compliance with PAP therapy and have a positive effect on the use of the PAP device. Our previous data showed that high-grade septal deviation and inferior turbinate hypertrophy correlate with low PAP compliance and suggested additional therapeutic approaches according to various anatomical characteristics^14^. In addition, other studies suggested the presence of AR and severity of nasal obstruction lead to a significant difference in the success rate of nasal surgery, and nasal surgery also reduces the nasal resistance, Epworth sleepiness scale score, and PAP pressure^31^. In concert with our clinical data mentioned above, it could be inferred that, if the MCA and NCV were smaller, the reduction in autoPAP pressure after surgery and the degree of subjective satisfaction improvement would be greater. The complaint of uncontrolled AR symptoms and blood eosinophil counts were significantly higher in group 2 after nasal surgery. Therefore, the high blood eosinophil counts might also be a good parameter to use to predict compliance with PAP therapy in OSA subjects who complained of nasal obstruction, and an evaluation for allergic diseases would be needed prior to prescribing PAP therapy to OSA subjects. Our clinical data did not prove the exact correlation between good compliance and the systemic eosinophil count, but the peripheral blood eosinophil count is known to be significantly correlated with the tissue-infiltrating eosinophil count and type 2 disease (including chronic rhinosinusitis and asthma) symptom aggravation, promoting airway edema^37–39^. The volatility of upper airway mucosal edema due to high type 2 inflammation might be a possible explanation for the absence of a decrease in PAP pressure after surgery and the poor compliance rate.

This study adds value to the field by investigating the factors that contribute to the reduction of PAP compliance in OSA subjects with nasal obstruction. The study considers both psychological and biomechanical factors, and demonstrates the necessity of nasal surgery for improved PAP device usage and compliance in those subjects. However, our study has several limitations. First, this retrospective case series lacks objective postoperative testing data. Additional data from postoperative PSG, rhinometry, and validated questionnaire with detailed items would be valuable in order to objectify the results. Second, only male subjects were included by coincidence, which may affect the generalizability of our results and require cautious interpretation. A large-scale prospective investigation of PAP compliance related to nasal surgery still needs to be performed in future studies.

## Conclusion

Our clinical study indicates that nasal surgery can be a beneficial surgical option for patients with obstructive sleep apnea (OSA) who experience nasal obstruction as a primary barrier to using continuous positive airway pressure (PAP) devices. Specifically, our findings suggest that nasal surgery may improve compliance with PAP therapy and lead to better treatment outcomes for OSA subjects with small nasal cavity volume and minimal allergic inflammation. However, it is important to note that nasal surgery may not be effective for OSA subjects who have multiple barriers to PAP device use or do not find PAP therapy to be effective. Further research is needed to confirm these findings and understand the underlying mechanisms.

## Supplementary Information

Supplementary Information.

## Data Availability

All the data generated and/or analyzed during the current study are included in this article and are available from the corresponding author on reasonable request.
