# Supplementary figures and images for: The clinical influence of nasal surgery on PAP compliance and optimal application among OSA subjects uncomfortable with PAP device wear
Source: Sci Rep. 2023 Mar 16;13:4383. doi: 10.1038/s41598-023-31588-7 (PMC10020433; doi:10.1038/s41598-023-31588-7)

Supplementary figure 1. The schematic flowchart of study design


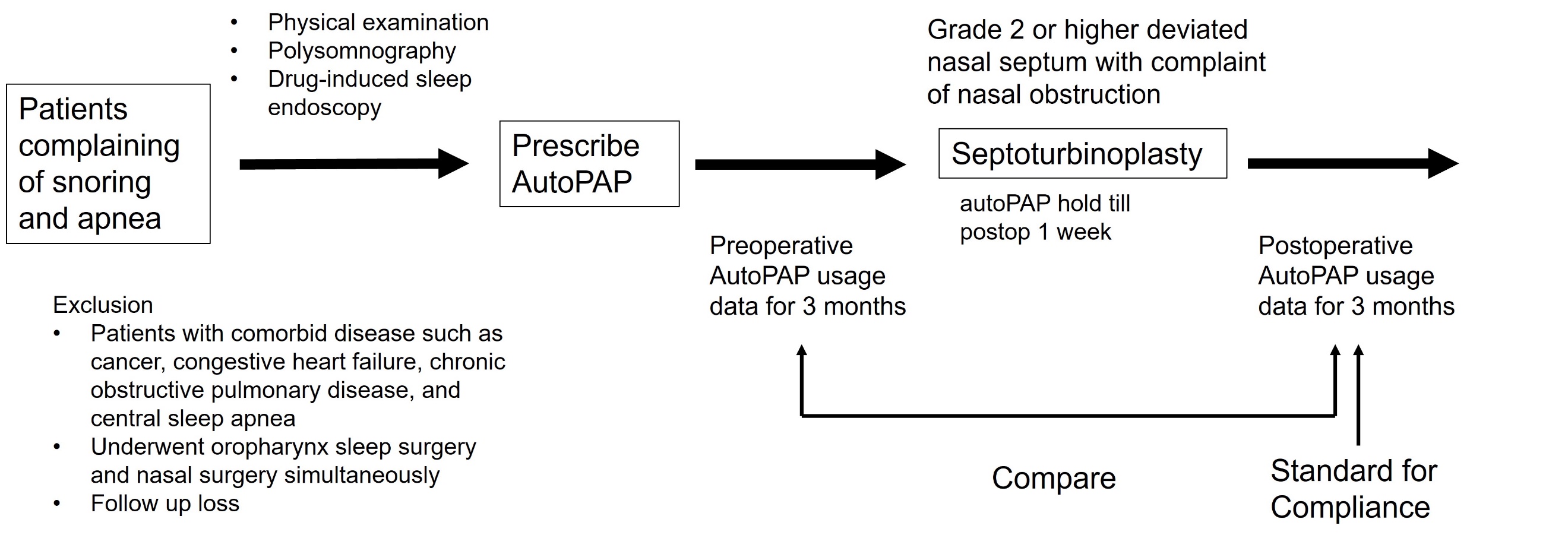

Supplement: Supplementary file 1 — Supplementary Information. [file 41598_2023_31588_MOESM1_ESM.doc]
